# Humoral Response after SARS-CoV-2 Vaccination in Prostate Cancer Patients

**DOI:** 10.3390/vaccines11040770

**Published:** 2023-03-30

**Authors:** Agata Błaszczuk, Dominika Sikora, Jacek Kiś, Ewa Stępień, Bartłomiej Drop, Małgorzata Polz-Dacewicz

**Affiliations:** 1 SARS Laboratory, Department of Virology, Medical University of Lublin, 20-093 Lublin, Poland; 2 1st Clinical Military Hospital with Outpatient Clinic in Lublin, 20-049 Lublin, Poland; 3 Department of Computer Science and Medical Statistics with the e-Health Laboratory, Medical University of Lublin, 20-090 Lublin, Poland

**Keywords:** COVID-19, prostate cancer, SARS-CoV-2 antibody, NCP, RBD, S2, vaccination

## Abstract

Cancer is an important public health problem. Prostate cancer is one of the most common cancers among men. In Poland, the incidence of this type of cancer is constantly growing. Considering the appearance of a new coronavirus in December 2019 (SARS-CoV-2) and the fact that oncology patients, including those with prostate cancer, are particularly vulnerable to infection, it is recommended to get vaccinated against COVID-19. In our study, we determined the level and prevalence of antibodies against SARS-CoV-2 IgG in patients with prostate cancer compared to the control group and whether the patients’ ages affected the level of antibodies. PCa patients and controls were divided into two age groups: 50–59 years and 60–70 years. We also analyzed the level of antibodies in patients belonging to the relevant risk groups for prostate cancer (the European Society of Urology risk group classification of prostate cancer). For the study, we used the Microblot-Array COVID-19 IgG test to detect antibodies against the three main SARS-CoV-2 antigens: NCP, RBD, and S2. Our results showed that prostate cancer patients had significantly lower levels of anti-SARS-CoV-2 IgG antibodies compared to controls. In addition, age also affected the decrease in the number of IgG antibodies. The level of antibodies in the intermediate/high-risk group was lower compared to the low-risk group.

## 1. Introduction

As of February 2023, over 757 million confirmed cases of COVID-19 and over 6.8 million deaths have been reported worldwide, with 6.4 million and 118,884 in Poland, respectively [1].

COVID-19 is a challenge in cancer care due to the risk of transmission and increased mortality in immunocompromised patients, especially in the elderly. Preventing infection and severe illness of COVID-19 is crucial for cancer patients, and vaccination is the most effective method to achieve this [2,3].

Prostate cancer (PCa) is one of the most common cancers among men. Worldwide, an estimated 1,414,259 people were diagnosed with PCa in 2020. It is the world’s fourth most commonly diagnosed cancer [4].

In Poland, the incidence of this type of cancer is constantly growing, and forecasts for Poland predict that this trend will continue.

According to the Polish Ministry of Health website and the National Health Fund, about 15,000 men are diagnosed with PCa every year, and we can probably expect about 20,000 cases a year soon. About 5000 men die yearly from PCa. The 1-year survival rate for men with prostate cancer is 92%, and the 5-year survival rate is 75.8% [5].

PCa is a multifactorial disease in which lifestyle, smoking, dietary habits, and family history play an important role [6].

Extensive studies on the molecular basis of various malignancies, including PCa, have revealed that the tumor formation process is affected by genetic and epigenetic alterations within the tumor cells and the rearrangement of the tumor microenvironment (TME) components. TME may differ in various tumor types, but generally, it consists of a cellular part containing tumor cells and stromal cells embedded in the ECM–a non-cellular component comprising, e.g., collagen, fibronectin, hyaluronan, and laminin. The interactions between all these elements are based on a complex network of cytokines, growth factors, inflammatory mediators, and matrix remodeling enzymes. They can promote the development and invasion of tumor cells. As ECM is a very dynamic structure, its components are still modified. Degradation of the ECM and basement membrane allows cancer cells to invade surrounding tissues and spread into blood vessels [7,8,9].

Degradation of the extracellular matrix (ECM) is required for tumor progression. Increased expression and/or activity of metalloproteinases (MMPs) play a key role in this process. The most studied is the secretion of MMP9 to promote cancer progression and metastasis [10].

Il-6 (proinflammatory cytokine) produced by inflammatory cells and osteoblasts is of great importance in the interaction with PCa cells by promoting cancer progression. Higher levels of this interleukin are observed in the serum of patients with metastases. It is through cytokines that host cells affect prostate cancer cells [11].

Some immune cells play a crucial role in TME and can have anti-cancer effects on the one hand and pro-cancer activity on the other [12].

The cellular components such as fibroblasts, T cells, tumor-associated macrophages (TAMs), and myeloid-derived suppressor cells (MDSCs) are cells that work together to secrete molecules that can mediate immunosuppression [11,12].

Macrophages that infiltrate the tumor tissue are termed TAMs. They produce a variety of pro-inflammatory factors that promote angiogenesis and tumor growth. The increased level of tumor-infiltrating macrophages is related to an unfavorable prognosis. Based on phenotypic differences, TAMs are divided into two subpopulations, M1-like and M2-like. M1-like macrophages typically participate in pro-inflammatory immune responses, while M2-like macrophages participate in anti-inflammatory ones [7].

The main feature of macrophages is their ability to phagocytize bacteria, viruses, etc. In addition, they are involved in cancer progression and metastasis. As the most numerous group of cells in the tumor microenvironment, they enhance cell migration, invasion, and epithelial–mesenchymal transition (EMT) [8].

Macrophages are one type of cell that significantly associate with tumor progression and immunosuppression [13,14]. In addition, crosstalk between PCa cells and infiltrating immune cells, including macrophages, neutrophils, T cells, B cells, and mast cells, affects immune cell function. All mentioned above, immune cells are significant in adaptive immunity [8].

T lymphocytes play a key role in the adaptive immune response. They are divided into many subtypes, including CD8+ T cells, CD4+ T cells, and regulatory T cells. CD 8+ T cells (cytotoxic T cells) directly kill virus-infected cells. CD4+ T cells (T helper cells) are responsible for the activation of B cells. Finally, regulatory T cells (Tregs) are essential for foreign antigen recognition [8].

B lymphocytes are essential for humoral response. The antibodies bind to the antigen and neutralize the microorganism. Then, B cells produce memory B cells, which provide a faster and stronger response to the same pathogen during reinfection. The presence of B cells in prostate TME may be associated with more aggressive disease [6,7,8].

As described above, the tumor immune microenvironment plays a significant role in the progression of prostate cancer. Therefore, Kwon et al. [8] proposed the use of immunogenic subtypes both in single and combination therapy, which may contribute to the improvement in prostate cancer treatment outcomes.

Due to weakened immunity, cancer patients are particularly vulnerable to infection, including SARS-CoV-2 [15,16,17]. Cancer patients were one of the first groups vaccinated against COVID-19. However, it is known that many people have recovered from COVID-19 despite being vaccinated [18].

Therefore, our study aimed to assess seroprevalence and antibody titers against SARS-CoV-2. Three types of antibodies were analyzed, i.e., nucleocapsid protein (NCP), S1 subunit receptor binding domain (RBD), and S2 protein subunit (S2).

The analysis was carried out depending on the patient’s age as well as the risk group. All results were compared with a control group of healthy men.

## 2. Materials and Methods

### 2.1. Study Design 

Our study included one hundred patients diagnosed with PCa histopathologically confirmed according to the European Society of Urology (EAU) classification [19,20,21].

The patients were hospitalized at the Urology Department of the 1st Clinical Military Hospital with Outpatient Clinic in Lublin. They underwent a radical prostatectomy. They had not received radiotherapy or chemotherapy before.

A control group consisted of 72 healthcare workers from our laboratory database with no chronic diseases. Men working in COVID-19 wards were excluded from controls.

The eligibility criterion for the study was the approximate age, vaccination against COVID-19 with two doses of Pfizer vaccine (the second dose of vaccine was administered no later than six months prior to the study), and mild history of COVID-19 without hospitalization (a documented positive RT-PCR test result from a nasopharyngeal swab confirmed COVID-19 infection). According to the recommendations of the Polish Ministry of Health, the second dose of the vaccine was administered no later than one month after the first dose. Mild infection with SARS-CoV-2 virus occurred after vaccination with the second dose, no later than six months after its administration.

PCa patients and controls were divided into two groups based on age: 50–59 years ($\bar{x}$ = 54) and 60–70 years ($\bar{x}$ = 67). All participants completed a questionnaire regarding demographic, epidemiological information, and prior exposure to COVID-19.

According to the EAU classification, there are three risk groups: the low-risk group, the intermediate-risk group, and the high-risk group, based on three data: prostate-specific antigen (PSA) level, Gleason score, and the TNM staging system [19,20,21]. This classification distinguishes the following risk groups: the low-risk group—PSA < 10 ng/mL, GS < 7 (ISUP grade 1), and cT1-2a; the intermediate-risk group—PSA 10–20 ng/mL, GS 7 (ISUP grade 2/3), or cT2b; the high-risk group—PSA > 20 ng/mL, GS > 7 (ISUP grade 4/5), or cT2c. We assigned PCA patients to those groups respectively. Finally, due to the small number of patients in the high-risk group, two groups were created for our analysis: the low-risk group and the intermediate/high-risk group.

### 2.2. Sample Collection

Venous blood samples (3–5 mL) from PCa patients were collected by qualified personnel in the laboratory of the 1st Clinical Military Hospital with Outpatient Clinic in Lublin. These blood samples were transported to our laboratory within 24 h at 3–5 °C and subjected to centrifugation (1500× *g* rpm/15 min at room temperature). The serum obtained after centrifugation was stored at −20 °C pending antibody analysis.

Based on our database (healthcare workers), we selected patients for the control group. The preparation and analysis of serum samples were described previously [22].

### 2.3. Detection of SARS-CoV-2 Antibody

Serum samples were analyzed using the Microblot-Array COVID-19 IgG assay kit (TestLine Clinical Diagnostics s.r.o., Brno, Czech Republic) for the detection of specific anti-SARS-CoV-2 antibodies (NCP, RBD, S2). This test detects the presence and titer of specific anti-SARS-CoV-2 IgG antibodies—NCP, RBD, and S2. Moreover, it allows the detection of cross-reactivity with other coronaviruses, i.e., Middle East respiratory syndrome coronavirus (MERS-CoV), severe acute respiratory syndrome coronavirus (SARS-CoV-2), human coronavirus 229E (HCoV 229E), and human coronavirus NL63 (HCoV NL63). The results are given in units of U/mL. The interpretation takes into account the presence or absence of a reaction against at least 1 antigen—NCP, RBD, or S2. The results were given in U/mL.

Reading and interpretation were performed using Microblot-Array reader and software: <185 U/mL = negative, 185–210 U/mL = borderline, >210 U/mL = positive.

### 2.4. Statistical Analysis

Results were analyzed using GraphPad Prism 9 software version 9.5.0. (San Diego, CA, USA). Categorical variables were expressed as counts and percentages. The prevalence of SARS-CoV-2 antibodies was reported as a percentage. The chi-squared test was used to compare prevalence of antibodies in both groups. The Shapiro–Wilk test was used to test the normality of continuous variables. The Mann–Whitney U test was used to analyze the antibody level depending on age and risk groups of the EAU classification. A *p*-value of <0.05 was considered to show a statistically significant result.

### 2.5. Ethics

The research was approved by the Medical University of Lublin Ethics Committee and is in accordance with the GCP regulations (no. KE-0254/194/10/2022, 10 October 2022). Written informed consent was obtained from each participant.

## 3. Results

### 3.1. Characteristics of the Studied Population

Details of the characteristics of PCa patients and controls are presented in Table 1.

PCa patients comprised 100 people, including 30% men in the 50–59 ($\bar{x}$ = 54) age group and 70% men in the 60–70 ($\bar{x}$ = 67) age group. Controls consisted of 72 healthcare workers, including 41.7% of men aged 50–59 and 58.3% of men aged 60–70 (Table 1).

Patients with PCa were also divided into cancer risk groups according to the EAU classification. Due to the small number of patients with PCa from the high-risk group, the patients for analysis were divided into the low-risk and the intermediate/high-risk groups. A total of 67.0% of patients were in the low-risk group, and 33.0% of patients were in the intermediate/high-risk group.

### 3.2. Prevalence of NCP, RBD, and S2 IgG Antibody in PCa Patients and Controls by Age

**Figure 1:**
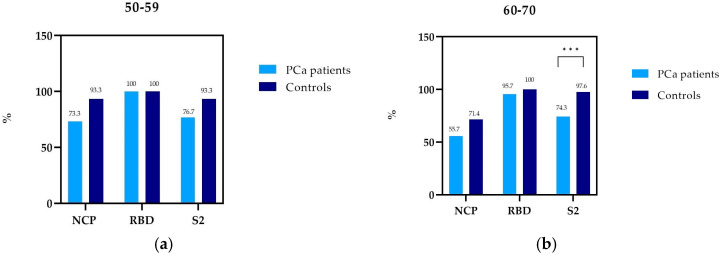
Prevalence of NCP, RBD, and S2 IgG antibodies in PCa patients and controls by age: (**a**) 50–59, (**b**) 60–70; *** statistically significant (10^−3^).

In the 50–59 age group, NCP antibodies were detected in 73.3% of patients with PCa, while in controls, 93.3% (Table 2, Figure 1). It was similar in the 60–70 age group, where NCP antibodies were detected more often in controls (71.4%) than in PCa patients (55.7%).

In both study groups, RBD antibodies were present in 100.0% of patients aged 50–59. In PCa patients in the 60–70 age group, RBD antibodies were detected in 95.7%. This difference is probably due to the fact that a few patients (three people) with PCa did not develop antibodies after vaccination.

In the 60–70 age group, S2 antibodies were detected in 74.3% of patients with PCa and 97.6% of controls, which is a statistically significant difference.

PCa patients in the 50–59 age group produced fewer NCP and S2 antibodies compared to healthy subjects: NCP antibodies, 73.3% and 93.3%; S2 antibodies, 76.7% and 93.3%, respectively. The prevalence of RBD antibodies in PCa patients and controls was identical—100%. However, these differences in the prevalence of NCP and S2 antibodies in the 50–59 group were not statistically significant (Figure 1a). In the age group of 60–70, there was a difference in the prevalence of all tested antibodies in PCa patients compared to healthy people. Nevertheless, statistical significance was only observed with the prevalence of S2 antibodies (*p* = 0.0013) (Figure 1b).

Both PCa patients and healthy individuals in the age group of 50–59 appeared to produce more NCP antibodies compared to the respective groups aged 60–70. However, this difference was not statistically significant.

### 3.3. The NCP, RBD, and S2 IgG Antibody Levels in PCa Patients and Controls by Age

**Figure 2:**
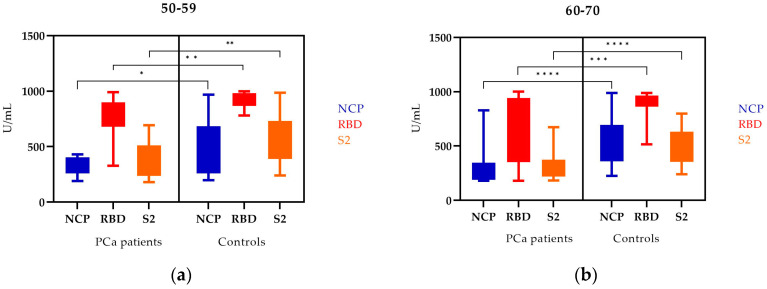
The levels of NCP, RBD, and S2 IgG antibodies in PCa patients and controls by age: (**a**) 50–59, (**b**) 60–70; Mann–Whitney U test; median (min–max); * statistically significant (10^−1^); ** statistically significant (10^−2^); *** statistically significant (10^−3^); **** statistically significant (10^−4^).

In the 50–59 age group, the levels of NCP and S2 antibodies (appearing after COVID-19) and the level of RBD antibodies were higher in controls. These differences were statistically significant (Figure 2a).

Analyzing the 60–70 age group, we also observed statistically significant differences in the levels of all types of antibodies. The levels were higher in controls than in PCa patients (Figure 2b).

A statistically significant difference was observed in NCP, RBD, and S2 antibody levels between PCa patients and controls for both age groups (Table 3).

### 3.4. NCP, RBD, and S2 IgG Antibody Levels in PCa Patients Depending on Age in Two Risk Groups (the EAU Risk Group Classification of PCa)

**Figure 3:**
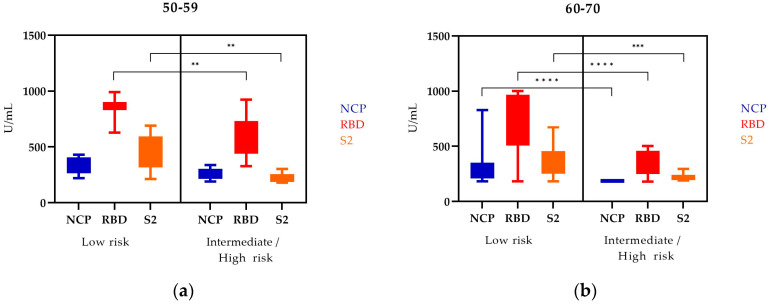
NCP, RBD, and S2 IgG antibody levels in PCa patients depending on age in two risk groups (the EAU risk groups): (**a**) 50–59, (**b**) 60–70; Mann–Whitney U test; median (min–max); ** statistically significant (10^−2^); *** statistically significant (10^−3^); **** statistically significant (10^−4^).

The levels of NCP, RBD, and S2 antibodies in PCa patients according to age and risk group are shown in Figure 3.

In the 50–59 age group, the levels of NCP, RBD, and S2 antibodies were higher in the low-risk group compared to the high-risk group. The differences in RBD and S2 levels in these patients were statistically significant.

The level of all tested antibodies in the 60–70 age group is statistically significantly higher in the low-risk group. Comparing both age groups, we observed that the levels of all tested antibodies were higher in the low-risk groups. However, we also noticed that the levels of NCP, RBD, and S2 antibodies were lower in the older age group compared to the younger group.

## 4. Discussion

Patients with chronic diseases, including cancer, are at high risk of developing a severe form of COVID-19. For this reason, vaccination against COVID-19 is especially recommended for them (also booster doses) [3,23,24].

Despite being vaccinated twice, many patients became infected with SARS-CoV-2, so they passed the infection relatively mildly, most often without requiring hospitalization. People who had a breakthrough infection acquired double immunity due to natural infection and vaccination (hybrid immunity) [18,19,20,21,22,23,24,25,26]. It would seem these people will have a higher humoral response in such cases. Better immunity is observed in healthy people [27].

However, cancer patients have reduced humoral and cell-mediated immunity. Moreover, these patients are very often burdened with other chronic diseases. Additionally used treatments, mainly chemo- and radiotherapy, affect the reduction in immunity.

One of the most common malignancies in men is PCa, which mainly affects older men, especially those over 60. It is a significant problem in developed countries where life expectancy has increased significantly. Because of their immunosuppressed status, PCa patients are more likely to be infected with SARS-CoV-2 [2].

Our research included a group of PCa patients who were vaccinated and yet had mild COVID-19 without hospitalization.

Immune response after vaccination against SARS-CoV-2 in patients with solid tumors has been described by many researchers [28,29].

Different researchers studied different groups of patients. Most studies are concerned with the assessment of seroprevalence of anti-SARS-CoV-2 IgG antibodies or antibody titers. Some studies have assessed the dynamics of antibody levels after one, two, or more doses of the vaccine [30].

We assessed both the frequency and the levels of antibodies against three antigens, i.e., NCP, RBD, and S2. A lower prevalence of individual types of antibodies was observed in PCa patients than in the healthy group.

Similar results were obtained by other authors. Oliver Overheu et al. [31] observed a low seroprevalence of SARS-CoV-2 antibodies among oncological patients in Germany after SARS-CoV-2 infection, and therefore, vaccination was highly recommended.

Many authors have performed meta-analyses. A meta-analysis by Cavanna et al. [32] showed that the prevalence of antibodies in cancer patients, especially with solid tumors, was high (90%). However, they had significantly lower antibody titers compared to healthy individuals. Ainsley Ryan Yan Bin Lee et al. [33] presented similar results in their meta-analysis. Martins-Branco et al. pointed out that the seroprevalence rate in patients with solid tumors was 94% (95% CI, 88–97%) [34]. An important consequence of immunosenescence is an impaired response to vaccination, characterized by reduced titers of vaccine-specific antibodies, shorter duration of measurable vaccine-specific antibodies, and reduced quality and affinity of antibody responses [35,36].

Many studies by other authors showed that cancer patients had a reduced humoral response after receiving a double dose of the COVID-19 vaccine compared to healthy individuals [37,38,39,40,41].

The present study demonstrated lower levels of all types of antibodies in PCa patients compared to controls. The lower level was observed both in the younger (50–59 years) and older groups (60–70 years). The studies of other authors also confirmed that the patient’s age is an additional factor in lowering immunity. One of the consequences of aging is the gradual dysregulation of the immune system [42,43,44].

Moreover, we observed that patients with advanced PCa had lower antibody levels. So far, there have been no studies on this subject, considering the division of patients into risk groups and the analysis of anti-SARS-CoV-2 antibodies produced by them. Probably, patients at a high risk of PCa have weaker immunity and, hence, lower antibody levels.

In cancer patients, most vaccines are effective in producing strong antibody responses when more than one dose is given. The more doses, the higher the serological response is [45,46]. It is suggested that seronegative patients should get booster vaccines. Regular booster doses may be effective in immunocompromised cancer patients. In addition, high vaccination rates in the community, especially among families of vulnerable patients and in healthcare settings, will help protect those with impaired responses to vaccination [33,46,47,48]. The research results by Ainsley Ryan Yan Bin Lee [33] showed the importance of the second dose of the COVID-19 vaccine and the subsequent third dose of the vaccine (booster), especially for immunocompromised patients. The second dose of the vaccine was associated with significant improvements in seroconversion and antibody titers.

Compared to vector vaccines, a better response to mRNA vaccines has been observed [49]. Furthermore, humoral immunity appears stronger after mRNA vaccination, while cell-mediated immunity is stronger after vector-based vaccines [50].

All participants in our study were vaccinated with the mRNA Pfizer vaccine.

Vaccination reduces but does not eliminate SARS-CoV-2 transmission. There is an increase in the incidence of SARS-CoV-2 breakthrough infections among vaccinated persons [51,52,53].

Currently, the subvariant, omicron, and its subsequent variants are circulating in the population. In addition, an assessment of vaccine effectiveness against different strains is hampered by the rapid emergence of new variants of concern [54]. Therefore, it is necessary to constantly monitor the changing immunity in order to develop the best prevention strategy.

Patients who qualified for our study did not undergo radio-, chemo-, or steroid therapy. All our PCa patients underwent radical prostatectomy, which may have affected their lowered immunity. On the one hand, it should be emphasized that patients who are treated with anti-cancer therapy show reduced humoral immunity after a breakthrough infection. On the other hand, Cohen et al. noted a significantly higher level of SARS-CoV-2 antibodies among people after breakthrough infection than in those vaccinated four times [55]. Both surgical stress and drugs used for anesthesia and analgesia reduce immunity. During this period, patients are at risk of developing postoperative infections and sepsis [56]. Therefore, prophylactic antibiotic therapy is used. This may have contributed to the reduced immunity of the patients we studied, even though they did not have anti-cancer therapy.

A limitation of our study was the small number of patients, especially regarding PCa risk (risk groups). We were unable to assess accurately how the degree of risk affects immunity. The limited follow-up period of the patients did not allow us to track the dynamics of the patients’ antibodies over time, that is, how long the antibodies persisted in PCa patients compared to healthy men. We analyzed only the humoral response. It would be worth assessing the cell-mediated response, which may be the subject of further research. It would be interesting to include other markers to determine whether these untreated patients were immunocompromised. Further research is also needed to develop the best preventive strategy for patients with solid organ cancer.

## 5. Conclusions

The prevalence of all types of antibodies in PCa patients than in healthy ones was lower—statistically significant differences concerned only S2 antibodies. PCa patients had significantly lower levels of anti-SARS-CoV-2 IgG antibodies compared to controls. In addition, age also affected the decrease in seroprevalence. The intermediate/high-risk PCa patients indicated worse humoral immunity compared to the low-risk patients regardless of age.

## Figures and Tables

**Table 1:** Characteristics of PCa patients and controls.

Parameters		PCa Patients		Controls	
		*n*	%	*n*	%
Age	50–59	30	30.0	30	41.7
	60–70	70	70.0	42	58.3
Risk	Low	67	67.0	-	-
	Intermediate/high	33	33.0	-	-

*n*—number of patients.

**Table 2:** Prevalence of NCP, RBD, and S2 IgG antibodies (U/mL) in PCa patients and controls by age.

	NCP				RBD				S2			
	PCa Patients		Controls		PCa Patients		Controls		PCa Patients		Controls	
	*n*	%	*n*	%	*n*	%	*n*	%	*n*	%	*n*	%
50–59	22	73.3	28	93.3	30	100.0	30	100.0	23	76.7	28	93.3
*p*	0.0797								0.1455			
60–70	39	55.7	30	71.4	67	95.7	42	100.0	52	74.3	41	97.6
*p*	0.1119				0.2905				0.0013 *			

* Statistically significant.

**Table 3:** The levels of NCP, RBD, and S2 IgG antibodies (U/mL) in PCa patients and controls by age.

	NCP		RBD		S2	
	PCa Patients	Controls	PCa Patients	Controls	PCa Patients	Controls
50–59	298.0	461.3	877.8	962.7	335.6	501.8
	(190.1–430.5)	(196.3–969.1)	(326.3–990.8)	(780.0–999.9)	(180.7–691.6)	(239.9–986.5)
*p*	0.0157 *		0.0018 *		0.0023 *	
60–70	217.1	545.1	696.1	917.4	266.1	476.1
	(179.6–827.9)	(225.6–989.2)	(180.0–1000.0)	(515.1–989.9)	(182.1–672.2)	(239.9–797.5)
*p*	<0.0001 *		0.0005 *		<0.0001 *	

Mann–Whitney U test; median (min–max); * statistically significant.

## Data Availability

The data presented in this study are available in the article.
